# To the Editors of the Medical and Physical Journal

**Published:** 1800-10

**Authors:** A. Willson

**Affiliations:** Montrose, North Britain


### *To the Editors of the Medical and Phyfical Journal.*

GENTLEMEN,

IF the information of an individual practitioner, far removed from the metropolis, can add any thing to the very high medical authority with which you have favoured the public on the fubject of the Vaccine Inoculation, my teftimony is very much at your fervice; it may, in fome meafure, ferve to afcertain the progrefs of that very ufeful difcovery all over this ifland.

In the month of July laft, during our warmeft weather, the confluent Small-pox appeared in the neighbourhood of fome of the firft families in this part of the country, who became very much alarmed for the fafety of their children. I was confulted, and on due enquiry found moft of the children under very unfavourable circumftances to receive the variolous infection; I mentioned my objections to the parents, and at the fame time propofed the Vaccine Inoculation, under the complete impreffion it would be equally fuccefsful in refifting contagion. My propofal was readily agreed to; Vaccine matter was immediately procured; the children were inoculated, and took the infection in the moft diftinct and favourable manner; the fears of the parents were completely removed; and from this fuccefs, and the example of the firft families, I am happy to inform you, that the Vaccine Inoculation is becoming general in this diftrict of country. I am,

GENTLEMEN,

Your obedient humble fervant,

*Montrose, North Britain,*

*Sept.* 10, 1800.

A. WILLSON, M. D.

